# Probiotic and Antioxidant Potential of *Lactobacillus reuteri*LR12 and *Lactobacillus lactis*LL10 Isolated from Pineapple Puree and Quality Analysis of Pineapple-Flavored Goat Milk Yoghurt during Storage

**DOI:** 10.3390/microorganisms8101461

**Published:** 2020-09-23

**Authors:** Naif Abdullah Al-Dhabi, Mariadhas Valan Arasu, Ponnuswamy Vijayaraghavan, Galal Ali Esmail, Veeramuthu Duraipandiyan, Young Ock Kim, Hyungsuk Kim, Hak-Jae Kim

**Affiliations:** 1 Department of Botany and Microbiology, College of Science, King Saud University, P.O. Box 2455, Riyadh 11451, Saudi Arabia; naldhabi@ksu.edu.sa (N.A.A.-D.); mvalanarasu@ksu.edu.sa (M.V.A.); gesmail@ksu.edu.sa (G.A.E.); avdpandiyan@gmail.com (V.D.); 2 Bioprocess Engineering Division, Smykon Biotech Pvt Ltd, Nagercoil, Kanyakumari District, Tamil Nadu 629 001, India; venzymes@gmail.com; 3 Department of Clinical Pharmacology, College of Medicine, Soonchunhyang University, Cheonan, Chungcheongnam 31151, Korea; kyo9128abcd@gmail.com; 4 Department of Rehabilitation Medicine of Korean Medicine, College of Korean Medicine, Kyung Hee University, Seoul 02447, Korea; kim0874@hanmail.net

**Keywords:** pineapple puree, lactic acid bacteria, yoghurt, post-fermentation, stable flavor

## Abstract

In recent years, studies have focused on the therapeutic properties of probiotics to eliminate pathogenic microorganisms associated with various diseases. Lactobacilli are important probiotics groups that have been found to possess many health-promoting activities. This study was carried out to isolate *Lactobacillusreuteri*LR12 and *L. lactis*LL10 from pineapple puree. The invitro analysis to evaluate probiotic characteristics of the isolated bacteria included survival in bile and acid tolerance. The cell-free supernatant of *L. reuteri* LR12 was effective against various pathogenic bacteria and fungi compared with *L. lactis*LL10. These two bacterial strains have strong anti-biofilm activity (100%) against *Enterococcus faecalis*, *Staphylococcus aureus*, and *Bacillus cereus*. The bacterial strains exhibited adhesion properties to HT-29 cells (human colorectal adenocarcinoma). These bacteria showed DPPH- (2,2-diphenyl-1-picryl-hydrazyl-hydrate) free radical scavenging activity, scavenging of hydroxyl radical activity, superoxide radical scavenging activity, and reducing power activity in the range of 72% ± 3%to 89.3% ± 1.7%, 64% ± 2.7%to 66.8% ± 1.5%, 59.8% ± 4.1% to 63.8% ± 2.1%, and 60.4% ± 1.8%to 66.1% ± 3.3%, respectively. Pineapple puree was used as the starter culture with milk for 2 days for yogurt preparation. Pineapple puree increased flavor and showed the physicochemical properties of yogurt. The finding of the sensory evaluation revealed no significant change compared with the control, except the appearance of yogurt. These findings show that Lactobacilli and pineapple puree have potential use in various probiotic preparations for the fermentation industry.

## 1. Introduction

Intestinal microbial flora intertwines metabolic and signaling capabilities to provide various health benefits. In recent years, studies have focused on investigating appropriate therapeutic agentsfor their antibiotic potential and ability to eliminate multidrug-resistant (MDR) microorganisms [1]. Biofilm-producing *Enterobacteriaceae* and carbapenemase producing *Klebsiellapneumoniaea* are prevalent in gastrointestinal tract infections. MDR bacteria cause intestinal sepsis, which is a serious health condition. In spite of prophylactic measures and appropriate antibiotic administration to the individuals, increases in antibiotic exposure pose serious risks to antibiotic resistance. Most of the bacteria of Gram-negative type are resistant to ampicillin, cloxacillin, and gentamicin [2]. Gastrointestinal infections are very common among infants [3]. Superoxide anion radicals, hydrogen peroxide, and hydroxyl radicals have been reported as reactive oxygen species (ROS), but hydrogen peroxide is only one of the hyperactive oxygen free radicals. These byproducts are involved in a number of events. In the human body, free radicals are involved in oxidative stress [4]. Many factors, namely nitrogen oxides, herbicides, ozonization, radiation, and some metals, induce oxidative stress. In phagocytosis, bacteria are invaded, which stimulates the production of reactive oxygen species due the activity of NADPH oxidase, and this enzyme is also involved in phagocytes [5]. In living organisms, enzyme defenses and non-enzymatic antioxidant defenses are reported. Enzymes like glutathione reductase (GR), glutathione peroxidase (GPx), and superoxide dismutase (SOD) are involved in phagocytosis, whereas non-enzymatic substances like vitamin E, vitamin C, thioredoxin, and glutathione are involved in the antioxidant defense system [6]. Despite several known synthetic antioxidants, including butylatedhydroxyanisole and butylatedhydroxytoluene, involved in treating free radicals, investigating suitable, natural, and safer antioxidants from natural resources has received considerable attention [7]. In recent decades, several works have been carried out on the probiotic potential of microorganisms, because these microorganisms have numerous health benefits, including antimicrobial and antioxidant activities [8,9,10]. Probiotics are living microbial food supplements and produce various beneficial biomolecules, including bacteriocins [11]. Probiotics add critical value to certain functional foods. Lactobacilli are important probiotics strains found to possess many health-promoting activities, as well as a long, safe history of being consumed by human beings [12]. For example, *Lactobacillus acidophilus* ATCC 4356 shows strong antioxidant activity and the capability of inhibiting linoleic acid. These strains survive and colonize the gastrointestinal tract of the host organism. The survival ability of probiotic organisms in the gastrointestinal tract is mainly influenced by the buffering capacity of the supplemented food. Yogurt, cheese, and skimmed milk are formulated with pH ranging from 3.5 to 4.5 and high buffering capacity, which increase the pH of the gastrointestinal tract and thus enhance the stability of probiotic strains [13]. In recent years, research has focused on the isolation of new bacterial strains with beneficial effects that mainly constitute natural inhabitants in the gastrointestinal tract. For example, the beneficial effect of *Akkermansiamuniciphila* with respect to gut modulation and immune modulation has been analyzed [14]. The selected bacterial strains must survive food processing and food storage conditions and withstand various stress conditions encountered in the upper gastrointestinal tract of the host organism. Hence, the suitable selection of probiotic bacteria and their potential delivery remains a challenge, with the main focus on sustaining the viability of the probiotic bacteria in the final formulated food product. Food and food products are widely used for the isolation of Lactobacilli. The pineapple fruit is an edible fruit made of flesh, with simple sugars like fructose and sucrose. This fruit is rich in antioxidants (vitamin C), and consumption of this fruit is beneficial against various organisms and scavenging activity [15]. Fermentation of pineapple puree and the survival of probiotic bacteria in pineapple juice are not very clear. In this study, two *Lactobacillus* sp. were isolated from pineapple puree, and biological activities were studied. Then, pineapple puree was used as the starter culture for goat milk yogurt production for nutraceutical applications.

## 2. Materials and Methods

### 2.1. Pineapple Puree Preparation

A freshly collected pineapple was used for the preparation of pineapple puree. About 500 g of pineapple pulp was prepared by using 50 g fruit body ground with double-distilled water and was autoclaved. It was transferred into an Erlenmeyer flask containing 200 mL of raw milk. In this mix, gelatin (2%, *w*/*v*), sugar (2%, *w*/*v*), and salt (0.8%, *v*/*w*) were incorporated and stirred continuously. The mixture was then dispensed into a sterile container and kept at 37 °C for 72 h. The stability of the sample was analyzed continuously and was used for the isolation of Lactobacilli. 

### 2.2. Isolation, Screening, and Characterization of Lactobacillus Strains

About 0.1 mL of pineapple puree sample was placed on MRS (Man, Rogosa, Sharpe) agar containing CaCO_3_ (Himedia, Mumbai, India) and further incubated at 30 ± 2 °C for 72 h under anaerobic state. The bacterial isolates were selected based on acid production (clear zone around the colony) on MRS medium containing CaCO_3_ [16]. Acid-producing organisms were sub-cultured prior to use in MRS liquid medium followed by incubation at 37 °C for 72 h. The growth of the bacterial strains was measured at 600 nm using a UV-visible spectrophotometer. The bacterial strains (LR12 and LL10), which showed maximum cell density (1.090 ± 0.1 and 1.048 ± 0.1 OD [optical density]) at 600 nm, were subjected to morphological, biochemical, and *16S* rDNA sequencing.

### 2.3. LAB Strain and Resistance

The antibiotic resistance pattern of *Lactobacillus* (LAB) strains (LR12 and LL10) was evaluated using the disc diffusion method. In this method, about 100 µL of *Lactobacillus* strains (LR12 and LL10) were placed on MRS agar medium, and then the commercial antibiotic discs were placed. The culture plates were incubated for 24 h at 30 ± 2 °C, and antibiotic resistance was determined [17].

### 2.4. Probiotic Characterization of LAB

To analyze the acid tolerance of LAB strains (LR12 and LL10), MRS medium was preparedwith3% bile salts. Then, 100 μL of MRS broth (log phase) was inoculated and incubated at 37 °C for 24 h, and its tolerance level was evaluated. To evaluate acid tolerance, 3% oxgall acid was added to the MRS broth medium and inoculated at the log phase of the bacterial strain. The culture was incubated at 37 °C for 24 h, and the oxgallic acid tolerance (%) was evaluated. The viability of bacteria was calculated after serial dilution and growth on MRS agar medium. Acid and bile tolerance experiments were repeated three times, and an average value was considered for analysis [16]. The survival rate was determined using the given formula:

Survival rate (%) = (cell no. after reaction (CFU)/initial cell no. (CFU)) × 100

### 2.5. LAB Strains and Adhesion Properties

To evaluate the adhesion properties of *Lactobacillus* strains (LR12 and LL10) on HT-29 cells (intestinal epithelial cells), cells were added to a 24-well microtiter plate at 2 × 10^5^ cell concentration and incubated for 24 h at 37 ± 2 °C. On the developed HT-29 monolayer, *Lactobacillus* strains (LR12 and LL10) were seeded at 1 × 10^8^ CFU/well. The microtiter plate was incubated for about 2 h at 37 ± 2 °C with 5% CO_2_ using a CO_2_ incubator. Further, the non-adherent HT-29 cells were removed using PBS (Phosphate Buffer Saline) by repeat washing. Then, 0.1% Triton X-100 was used on each well, and bacterial cells were harvested. The cells were then serially diluted and spread on MRS agar plates [18]. The plates were incubated for 24 h at 37 °C, and adhesion properties were calculated as below:

Adhesion ability (%) = (adhered cell no. (CFU)/initial cell no. (CFU)) × 100

### 2.6. Test Organisms for Antimicrobial Assay

*Escherichia coli* (ATCC 8739), *Pseudomonas aeruginosa* (ATCC 15442), *Salmonella typhi* (ATCC 13311), *Bacillus cereus* (ATCC 14579), *Enterococcus faecalis* (ATCC 29212), and *Staphylococcus aureus* (ATCC 6538) bacteria were used for analysis. *Aspergillus niger* (ATCC 16404), *A. flavus* (ATCC 9643), *A. nidulans* (ATCC 38163), and *Penicillium expansum* (ATCC 7861) fungal species were also tested. Bacterial strains were cultured in nutrient broth medium (Himedia, Mumbai, India) for 18 h at 37 °C. The fungal strains were cultured in potato dextrose broth for 72 h at 37 °C. The growth of culture was observed using a UV-visible spectrophotometer at 600 nm.

### 2.7. Preparation of Cell Free Supernatant

The Lactobacilli strains (LR12 and LL10) were cultured individually in MRS broth (Himedia, Mumbai, India) medium for 24 h at 37 °C. The culture was centrifuged at 10,000 rpm for 20 min at 4 °C. The supernatant was filter sterilized using a 0.22 μm filter, as described earlier [19].

### 2.8. Antibacterial Susceptibility Testing of LAB

To determine antagonistic activity, the disc diffusion method was followed. The discs were prepared with Whatman’s filter paper (no. 1) with a diameter of 6mm. The paper was sterilized, and the prepared disc was aseptically soaked into the cell-free supernatant of LAB. Sterile Mueller–Hinton agar (MHA) was aseptically poured into sterile Petri dishes, and the overnight culture of test organisms was inoculated. Then, sterile forceps were used to place the disc aseptically on the surface of MHA plates. Ciprofloxacin was used as a positive control, and the plates were incubated for 24 h at 37 °C. To study the influence of organic acid of the cell-free extract on antimicrobial activity, the pH of the filter sterilized supernatant was adjusted to 6.5 by using 1 N NaOH [20]. The plates were observed for zones of inhibition (mm).

### 2.9. Antifungal Activity of Lactobacillus Strains

Antifungal activity was evaluated using the agar diffusion method, as suggested by Arasu et al. [21]. LAB strains were streaked on the plates containing 25 mL MRS agar, and the plates were incubated for three days at 37 °C. About 50 µL of fungal suspension with 10^8^ CFU/mL was incorporated into the PDA medium. It was overlaid on MRS agar solid medium with the *Lactobacillus* strains LR12 and LL10. The culture plates were incubated for three days at 37 °C, and the zone of inhibition was analyzed.

### 2.10. Minimum Inhibitory Concentration

Minimum inhibitory concentrations (MICs) of both bacteria and fungi were evaluated separately by the tube dilution method. To determine the MIC of bacteria and fungi, one milliliter of cell-free extract of LAB was serially diluted up to a predetermined concentration. All tubes were inoculated with fungi (10^8^ CFU/mL) and bacteria separately. The antifungal agent ketoconazole was used as standard. The MIC was analyzed as the lowest quantity of the cell-free extract that completely inhibited the growth of the organisms.

### 2.11. Determination of Minimum Bactericidal Concentration

To evaluate the minimum bactericidal concentration (MBC), tubes without any sign of turbidity/growth in MIC samples were cultured on nutrient agar medium and incubated for 24 h at 37 ± 2 °C. The lowest concentration of cell-free extract of LAB that effectively inhibited growth was calculated as the MBC.

### 2.12. β-Glucuronidase Assay

The selected bacterial strains (LR12 and LL10) were cultured in MRS liquid medium at 37 °C. After 18 h of incubation, the bacterial cells were centrifuged at 5000× *g* for 10 min, washed three times with PBS, and mixed with lysis buffer (acetone/toluene, 9:1 *v*/*v*). Then, an 80 μL aqueous layer was separated and mixed with ρ-nitrophenyl-β-D-glucuronide (5 mM) and kept for 37 °C at 30 min. The enzymatic reaction was stopped by adding 0.5 M Na_2_CO_3_, and the released pNP was evaluated using a microtiter plate reader at 405 nm [22].

### 2.13. Biofilm Inhibition Assay

A biofilm inhibition assay was performed using biofilm forming *E. coli*, *P. aeruginosa*, *S. typhi*, *B. cereus*, and *E. faecalis*, as described by Wu et al. [23]. These bacterial strains were prepared at 1 × 10^6^ CFU/mL concentration and inoculated in brain–heart infusion (BHI) broth (Himedia, Mumbai, India), and culture extracts of the *Lactobacillus* strains (LR12 and LL10)were separately added at 35 mg/mL concentrations. The microtiter plate was incubated for about 18 h and, the non-adherent cells were removed using sterile double-distilled water. Then the adherent bacterial cells were fixed by using 250 µL methanol for 10 min, and the plates were air-dried. The fixed biofilms were stained by using crystal violet (0.2%, 300 µL) prepared in double-distilled water. The crystal violet stain of adherent cells was extracted using glacial acetic acid (33%, 200 µL). The absorbance of the sample was read at 540 nm, and biofilm inhibition efficiency was analyzed.

Biofilm inhibition rate (%) = (1 − (absorbance of sample)/(absorbance of control)) × 100

### 2.14. Inhibition of Glucan Synthesis

The bacterial test strain was inoculated in BHI broth and incubated for 24 h at 37 °C. It was further centrifuged (at 5000× *g*), and the aliquots were kept for 15 min at 4 °C. The sample was filtered using a membrane filtration unit (0.2 μm membrane filter). Then 20 μL of crude sample was transferred in a reaction mixture containing 180 μL of LAB supernatant and potassium phosphate buffer (62.5 mM, pH 6.5) containing 0.25 g of sodium azide and 12.5 g of sucrose, and incubated for 30 h at 37 °C. The adhered sample with tubes were collected and sonicated for complete dispersion. Finally, the amount of water-insoluble glucan was calculated by measuring the sample at 550 nm [24], and the glucosyltransferase (GTF) inhibition rate was determined as described below:

GTF inhibition rate (%) = (1 − (absorbance of treated sample)/(absorbance of control)) × 100

### 2.15. In Vitro Antioxidant Activity of LAB Strains

#### 2.15.1. DPPH Free Radical Activity

Briefly, 12.5, 25, 50, and 100 µL extracts of *Lactobacillus* isolates (LR12 and LL10) were added into DPPH radical solution (0.05 mM). The solutions were mixed and kept in the dark for 30 min [25]. The absorbance samples were measured at 517 nm. The scavenging ability was calculated as below:

Scavenging ability (%) = (1 − *A_sample_* − *A_blank_*)/*A_control_*) × 100

#### 2.15.2. Scavenging of Hydroxyl Radicals

Scavenging activity of hydroxyl radicals by bacteria was measured according to the method of Guo [13]. Overnight, bacterial samples with different concentrations (12.5, 25, 50, and 100 µL) were inoculated into samples containing O-phenanthroline (0.1%, *w*/*v*), 2.5 mM FeSO_4_, and 20 mM H_2_O_2_. The samples were then incubated for 90 min at 37 °C. The absorbance samples were read at 536 nm, and free radical scavenging activity was determined as follows:

Scavenging activity (%) = [(*A_1_* − *A_2_*)/(*A_1_* − *A_0_*)] × 100%

#### 2.15.3. Superoxide Radical Scavenging Activity

Samples were mixed at various concentrations (12.5, 25, 50, and 100 µL) with pyrogallic acid (0.05 M, 0.1 mL). The samples were incubated at 25 °C for 30 min in the dark. Absorbance samples was measured at 320 nm, and superoxide radical scavenging was analyzed [26] as follows:

Scavenging rate (%) = [1 − *A_320 nm sample_* − *A_320 nm blank_*] × 100%

#### 2.15.4. Reducing Power Assay

Samples at various concentrations (12.5, 25, 50, and 100 µL) were mixed with 1% potassium ferricyanide solution. The reaction mixture was incubated for 20 min at 50 °C. The mixture was cooled, and 10% trichloroacetic acid was added. The mixture was centrifuged, the upper layer was gently mixed with ferric chloride solution (0.1%), and the absorbance of the sample was analyzed at 700 nm [27].

### 2.16. Yogurt Preparation

#### 2.16.1. Yogurt Preparation Using Pineapple Puree

Goat milk was freshly collected and used for the preparation of yogurt. Yogurt preparation was based on the method suggested by Lee and Lucey [28], with few modifications. The collected milk was heated at 90 ± 2 °C for 5 min, and 5.0% (*w*/*v*) table sugar was added. The milk was then rapidly cooled to between 40 and 42 °C. Then, the starter culture (pineapple puree) was added at 1.0%, 2.0%, and 3.0% levels and incubated for two days for the process of forming yogurt. After 48 h of treatment, yoghurt was analyzed for various physicochemical factors and microbiological analyses. 

#### 2.16.2. Analysis of Physicochemical Parameters of Yogurt

The fat, ash content, acidity, pH, and dry matter of the yogurt were determined using the standard method [29]. Titratable acidic level and pH of the sample were determined after 2 days of yogurt preparation. The sugar content of yogurt was determined and expressed as % sucrose. The solid non-fat was also measured using the following formula:

Solid non-fat (%) = % dry matter − % fat content

#### 2.16.3. Analysis of Microorganisms and Sensory Evaluation of Yogurt

One gram of the yogurt sample was transferred into 99 mL sterile, double-distilled water, and samples were prepared at various dilutions (10^−1^ to 10^−7^). Total plate count agar was used for the determination of total bacteria population in the sample, whereas potato dextrose agar was used for the determination of total fungi. An appropriately diluted sample was spread on MacConkey Agar medium for the determination of total coliforms. All plates were incubated at 37 °C for 24 h for bacteria and 72 h for fungi. A colony counter was used for the determination of developed bacterial and fungal colonies. Twenty people were selected to evaluate the taste and flavor of pineapple yogurt. About 20 mL of product was served to all participants, and the results were registered. Clear instructions were given to the participants to clean their palates. Sensory scales were given to the participants to give ratings ranging from 0 to 10, with 0 being the lowest and 10 being the highest score in terms of taste. Sensory characteristics, such as appearance, texture, and flavor were improved in the puree-fermented yogurt [30].

### 2.17. Statistical Analysis

One-way analysis of variance was used to test the significance of variation in all experiments. A *p*-value of < 0.05 was considered as statistically significant.

## 3. Results and Discussion

### 3.1. Isolation of Probiotic *Lactobacillus* Strains

Most probiotic products have been used as functional food. In this study, two probiotic organisms were isolated from pineapple for the preparation of functional food, because the presence of high sucrose and fructose is preferred for the growth of *Lactobacillus* sp. Two probiotic isolates, *L. reuteri* LR12 and *L. lactis*LL10, exhibited weak resistance towards the antibiotics ampicillin, tetracycline, chloramphenicol, and doxycycline, while showing strong resistance against gentamycin, kanamycin, streptomycin, and ciprofloxacin. The findings of antibiotic susceptibility are highly similar to previous reports that have also shown the absence of strong resistance against various antibiotics from natural fermented sources [31]. Recently, probiotic bacterial strains from the genus *Enterococcus* were isolated from an Argentinean cheese, and their application as a starter culture for the production of cheese was suggested [32]. Probiotic bacteria were isolated from unfermented and fermented products of animal origin, such as honey, fish, seafood, and raw cured cold meats [33]. Recently, the probiotic properties of bacteria, such as *Lactobacillus fermentum* R6, *Lactobacillus curvatus* R5, *Lactobacillus brevis* R4, and *Pediococcuspentosaceus* R1 have been characterized [34].

### 3.2. Probiotic Properties of the LAB Strains

The tolerance effects of both *Lactobacillus* species were compared. When the bacteria were grown under 3% bile and 3% oxgall acid conditions, the survival power of *L. reuteri*LR12 was 85.3%, and the log CFU/mL value was 9.12 ± 0.07. The survival rate of *L. reuteri*LR12 was improved (100%) in the presence of bile salts. In *L. lactis*LL10, the initial log CFU was 8.65 ± 0.31/mL, and this value increased to 9.07 ± 0.77 log CFU/mL (Table 1). Cell count was also increased in the presence of bile salts (9.29 ± 0.15 CFU/mL). Bile-salt hydrolases (BSHs) showed adaption to the organism to survive in a bile-containing environment [35]. BSHs are inducible enzymes in *Lactobacillus*, and in *L. plantarum* expression of the bsh gene enhanced enzyme production over six-fold after exposure of this organism to 2% bile. BSH activities varied based on the source of the sample and host species. Increased levels of BSHs in a human host are related to a higher cholesterol-removing ability [36]. The finding suggested that enzymes of *Lactobacillu*s sp. play an important role for gut bacterial flora, mainly contributing to the ability of bile tolerance.

### 3.3. β-Glucuronidase Assay and Adhesion Ability

β-glucuronidase activity was analyzed, and it was found that the selected *Lactobacillus* strains (LR12 and LL10) did not produce this enzyme. β-glucuronidase activity is one of the important probiotic characteristics. In *Lactobacillus* species, β-glucuronidases liberate mutagens and toxins that are generally excreted with the bile juice. This process leads to elevated levels of carcinogenic compounds, thus enhancing the risk of cancer in the gut, as has been reported by Gill and Rowland [37]. In cancer patients, increased β-glucuronidase levels have been reported compared with normal individuals [38]. *L. reuteri*LR12 has a stronger adhesion ability (76.3 ± 1.93%) than *L. lactis*LL10 (71 ± 1.08%) (Table 1). Adhesion properties are an important characteristic of probiotics. Kos et al. [39] reported a correlation between hydrophobicity and adhesion ability in *Lactobacillus* strains. Colonization and adherence of *Lactobacillus* in the gut is a prerequisite for the isolates to exhibit various beneficial effects on humans [40]. Analysis of adhesions to the HT-29 intestinal epithelial cell line of *Lactobacillus* isolates has been considered as an effective in vitro method for screening isolates for probiotic potential [41]. Tuo et al. [42] reported adhesion properties and auto aggregation among various bacterial species, and positive correlations were observed between adhesion and auto aggregation.

### 3.4. Antimicrobial Activity of Lactobacillus Strains against Microbial Pathogens

The cell-free extract of the tested *Lactobacillus* strains (LR12 and LL10) showed potent antimicrobial activity. The zone of inhibition varied widely. *Lactobacillus* LR12 showed high potential activity against *S. aureus* (27 ± 1 and 26 ± 2 mm) and *E. coli* (28 ± 2 and 25 ± 1 mm) (Table 2). The selected *Lactobacillus* strains (LR12 and LL10) were analyzed to determine MIC against microbial pathogens, and the results are shown in Table 3. The cell free extract of *L. reuteri* (LR12) showed a very low MIC value against *P. aeruginosa* (6.25 µg/mL), *E. fecalis* (6.25 µg/mL) and *P. expansum* (6.25 µg/mL). The extract of *L. lactis*LL10 showed the lowest MIC value against *P. aeruginosa* (6.25 µg/mL) and *B. cereus* (6.25 µg/mL). The activities against bacterial pathogens were mainly due to the production of hydrogen peroxide, non-lactic acid molecules, and bacteriocin-like molecules [43]. Bacteriocins are enzymatic, degradable, proteinaceous compounds, and bacteriocins such as pediocin, nisin, and sakacin show potent antibacterial activity against bacterial pathogens [44]. The selected *Lactobacillus* sp. (LR12 and LL10) showed higher antifungal activities against *Penicillium* sp. than other fungal strains. This result is comparable with previous findings [21]. The antibacterial activity of *Lactobacillus* has been reported previously; for example, the reported *Lactobacillus* strains showed antibacterial activity against *C. difficile* [45], *E. coli* [46], *Shigella* spp. [47], *P. aeruginosa* [48], and *S. aureus* [49]. Recently, Wang et al. [50] isolated *L. plantarum* from Tibetan yaks and reported an inhibitory effect against *Staphylococcus aureus* and *E. coli*. The antibacterial activity of *Lactobacillus paracasei* subsp. *paracasei* was reported against *Staphylococcus aureus* involved in colonic and intestinal injury [51]. In this study, the sample showed high activity against *S. aureus*, and the neutralized cell-free extract did not show any antimicrobial properties. This revealed that the low pH value of cell-free extract due to the presence of organic acids, especially lactic acid produced by the selected Lactobacilli strains, is highly responsible for antimicrobial potential. Zhang et al. [52] also previously reported a loss of antimicrobial activity in the cell-free extract after neutralization.

### 3.5. Inhibition of Biofilm and Glucan Formation

The bacterial strains (LR12 and LL10) showed inhibitory properties against biofilm-forming bacterial pathogens. *L reuteri*LR12 showed s 53% ± 1.3% biofilm inhibition rate against *E. coli* and 68%± 3.2% against *P. aeruginosa*, whereas *L. reuteri* LR12 and *L. lactis* LL10 showed 68% ± 3.2% and 54% ± 1.5% activity against *P. aeruginosa*. These two LAB strains have strong biofilm activity (100%) against *S. aureus*, *E. faecalis*, and *B. cereus* (Table 4). The biofilm inhibition property of *Lactobacillus* has been previously reported by Melo et al. [53]. Some bacterial species have the ability to produce various substances with anti-adhesive properties. For example, *Lactobacillus fermentum* produced biosurfactant-like substances and inhibited biofilm formation of *S*. *mutans* [54]. In a study, a lipopeptide extracted from *Bacillus subtilis* showed anti-adhesive properties against *Staphylococcus aureus* [55]. According to the ionic characteristics of the hydrophilic region, the amphipathic molecules are classified as anionic, cationic, zwitterionic, and non-ionic substances. Among these, cationic amphiphilic molecules have the potential to inhibit bacterial biofilm. These amphiphilic molecules effectively prevent the aggregation of bacterial cells [56]. The present findings revealed the inhibitory effect of the biosynthesis of glucan from *S. aureus*. *L. reuteri*LR12 extract had an inhibition rate of 49.2% ± 1.7% and 33.5% ± 3.1% against *S. aureus* and *E. coli*, respectively. *L. lactis*LL10 extract showed the maximum inhibitory effect (40.2% ± 2.9%) against *S. aureus* (Table 5).

### 3.6. Antioxidant Activity of LAB

The selected *Lactobacillus* strains showed DPPH, hydroxyl radical, superoxide, and reducing power activities, and the results are described in Table 6 and Table 7. At 100 µL concentration, the sample from *L. reuteri* showed 89% ± 21% DPPH activity, 80% ± 3.2% hydroxyl radical antioxidant scavenging activity, 89 ± 3% superoxide radical antioxidant scavenging activity, and 90% ± 6.8% reducing activity. In *L. lactis*LL10, increased hydroxyl radical antioxidant activity was obtained for *L. reuteri*. In living organisms, an elevated level of oxygen radical byproducts can be obtained during the mitochondrial electron transport of aerobic respiration [57]. Many *Lactobacillus* strains show hydrogen peroxide antioxidant activity. Exopolysaccharides of *L. lactis* have exhibited promising antioxidant activity [58]. Certain *Lactobacillus* species have also degraded superoxide anions and hydrogen peroxidase [59]. The probiotic strains *Lactobacillus fermentum* E18 and *Lactobacillus fermentum* E3 have shown antioxidant properties [60]. A crude sample extracted from LAB strains showed antioxidant activities because of the presence of non-enzymatic substances and intracellular antioxidant enzymes [61]. In LAB, antioxidant enzymes, such as NADH oxidase, glutathione reductase, glutathione S-transferase, catalase, glutathione peroxidase, and feruloyl esterase, counteract oxidative stress [62,63]. The intracellular enzymes extracted from the bacterial cells by cell disruption showed antioxidant properties.

### 3.7. Changes inpH Value of Yogurt during Storage

**Figure 1:**
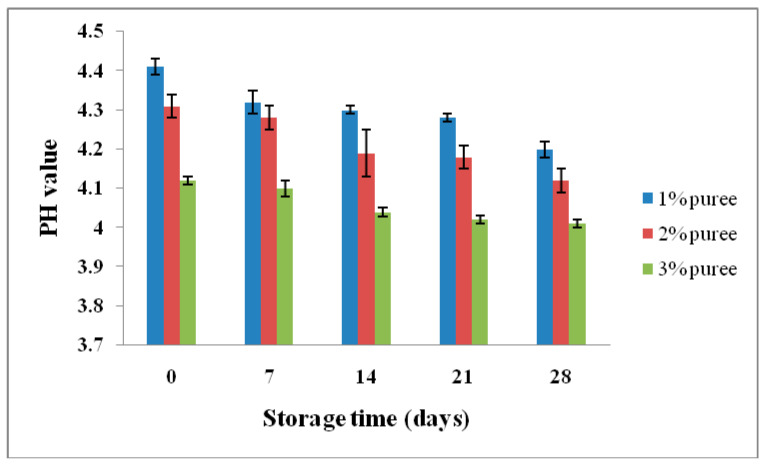
Influence of pineapple puree on goatmilk yogurt during storage.

The pH of the yogurt was analyzed for a period of 28 days, and a decrease of pH value was observed at 3% puree. However, this decreased level was not significant at 1% and 2% puree-inoculated yogurt (*p* > 0.05). Yoghurt with 3% puree-inoculated medium had a significantly reduced pH. The continuous decrease of pH value of the yoghurt indicated the activity of lactic acid bacteria at lower temperatures. However, the pH of the yoghurt changed based on the nutrient content and availability. The pH changes did not affect the quality and physical properties of yoghurt. The variations of pH value of yogurt for four weeks are shown in Figure 1. Nikoofar et al. [64] analyzed the pH value of yogurt prepared with quince seed mucilage. The pH value of the yoghurt decreased continuously during storage. The pH value decreased very little up to day 9, but a sudden decrease was observed at day 10. In the present study, the pH changes were similar to those observed in yogurts containing inulin [65] and waxy maize [66]. Thus, using pineapple puree in yogurt production had no significant negative impact on the bioprocess. In this study, the pH value of yogurt decreased at a higher percentage of puree-inoculated medium. Pineapple puree is rich in soluble carbohydrates, which support the growth of bacteria. LAB-synthesized lactic acid reduced the pH value of the yoghurt. The pH of the yoghurt declined based on the interactions between the nutrient composition of the pineapple puree and organic acid production. The decreased pH level under various storage conditions in relation to the starter culture and composition of fruit puree has been described previously [67]. Generally, addition of fruit pulp decreases the pH of yogurt, as reported previously [68].

### 3.8. Bacterial Population and Sensory Characteristics of Yogurt during Storage

Yogurt supplemented with 3% puree decreased the number of bacteria considerably compared with the 1% and 2% starter cultures. Supplementation of 1–3% puree reduced the total bacteria, and was mainly related to the antibacterial property of bromelain, an important component of pineapple. Yoghurt treated with 1% pineapple puree measured 15 × 10^2^ CFU/mL, and decreased to 10 × 10^−2^ CFU/mL when the yogurt was prepared using 3% puree (Table 8). The decreasing bacterial population was mainly due to the antibacterial activity of bromelain. The antibacterial activity of bromelain has been described previously [69]. In the yogurt, fecal coliforms were not detected. The microbial populationof yogurt from South Africa, Argentina, Greece, Norway, France, Spain, Australia, and the United States was examined, and the bacteria such as *L. delbrueckii* subsp. *Bulgaricus* and *S. thermophilus* were determined in the range of <10^4^ to 10^9^ CFU/mL. The population of *S. thermophilus* was higher than *L. delbrueckii* subsp. *bulgaricus*. The level of *Lactobacillus* spp. was about 10^8^ CFU/g [70]. In this study, the addition of puree did not affect the properties of yogurt. The evaluated sensory characteristics, such as appearance, texture, and flavor showed a moderate decreasing tend in all three treated yogurts (1%, 2%, and 3%). However, no significant difference was observed among experimental groups. During the post-fermentation storage or fermentation process, adjunct cultures or probiotics could mostly cause various complex chemical and physical changes, such as acidification and hydrolysis, as well as the formation of amino acids and other compounds. Within 28 days of storage in a refrigerator, the sensory properties were desirable for consumption. Recently, a *Bifidobacterium* monoculture was used to prepare yogurt from goat milk, and desired sensory characteristics were detected up to 21 days of storage [71]. Natural sources are rich in polyphenols and have antimicrobial and antioxidant activities [72]. Recently, Cheon et al. [73] reported probiotic and neuroprotective properties of *Lactobacillus buchneri* KU200793 isolated from Korean fermented foods. The inoculation of probiotics in food products requires specific techniques, as probiotic bacteria can experience stress during food processing and gastrointestinal transit [74]. Survival of bacteria in harsh environments is a prerequisite for the selection of probiotic bacteria. Most probiotic organisms show poor resistance to various technological processes, and this leads to limitations in the use of various food products. Improving bacterial resistance by adaptation or microencapsulation may allow these bacterial strains to be used to formulate varieties of functional food.

## 4. Conclusions

The present study investigates the effect of antioxidant and antimicrobial potential of *Lactobacillus* strains isolated from pineapple puree. Pineapple puree was used as the starter culture for the formation of yogurt from goat milk, and the properties of yogurt were investigated for 28 days of post-fermentation storage. Two *Lactobacillus* species, namely *L. reuteri* LR12 and *L. lactis* LL10, survived well in cold storage. The addition of pineapple puree up to 3% did not cause any physical and sensory changes compared to the control. The flavor and other physical properties were similar in yogurt inoculated with 1–2% pineapple puree. The flavor of pineapple puree mediated yogurt fermentation, and this process may have significant application in the dairy industry.

## Figures and Tables

**Table 1:** Probiotic characteristics of *Lactobacillus* (LAB) isolated from pineapple fruit.

Characteristics	*L. reuteri*	*L. lactis*
Cell count at initial stage (log CFU/mL)	9.27 ± 0.38	8.65 ± 0.31
Cell count at gastric conditions (log CFU/mL)	9.12 ± 0.07	9.07 ± 0.77
Cell count in bile salts (log CFU/mL)	9.32 ± 0.05	9.29 ± 0.15
β-glucuronidase activity (U/mL)	Not detected	Not detected
Adhesion ability (%)	71 ± 1.08	76.3 ± 1.93

Data expressed as mean ± standard deviation.

**Table 2:** Antimicrobial activity of *Lactobacillus* strains against bacteria and fungi.

Bacterial Strain	Zone of Inhibition (mm)	
	*L. reuteri*	*L. lactis*
*E. coli*	28 ± 2	25 ± 0
*S. typhi*	24 ± 1	23 ± 2
*P. aeruginosa*	26 ± 2	23 ± 1
*B. cereus*	24 ± 3	22 ± 1
*S. aureus*	27 ± 1	26 ± 2
*A. niger*	13 ± 1	21 ± 2
*A. flavus*	15 ± 0	16 ± 0
*A. nidulans*	10 ± 0	ND
*P. expansum*	ND	ND

ND: No results detected.

**Table 3:** Minimum inhibitory concentrations of the LAB cell-free supernatant against tested microbial strains.

Microorganisms	Minimum Inhibitory Concentration (MIC) (µg/mL)	
	*L. reuteri*	*L. lactis*
*P. aeruginosa*	6.25	6.25
*E. coli*	25.00	12.50
*S. typhi*	12.50	25.00
*S. aureus*	6.25	25.00
*E. faecalis*	12.50	12.50
*B. cereus*	12.50	6.25
*A. niger*	>25.00	12.50
*A. flavus*	>25.00	25.00
*A. nidulans*	12.50	25.00
*P. expansum*	6.25	12.50

**Table 4:** Biofilm inhibitory properties of *Lactobacillus* strains against bacterial strains.

Bacteria	Biofilm Inhibition Rate (%)	
	*L. reuteri*	*L. lactis*
*P. aeruginosa*	68 ± 3.2	54 ± 1.5
*E. coli*	53 ± 1.3	73 ± 3.6
*E. faecalis*	100 ± 0	100 ± 0
*S. aureus*	100 ± 0	100 ± 0
*B. cereus*	100 ± 0	100 ± 0

**Table 5:** Inhibitory property of LAB on glucan.

Bacteria	Inhibition (%)	
	*L. reuteri*	*L. lactis*
*P. aeruginosa*	28.2 ± 2.4	37.2 ± 2.6
*E. coli*	33.5 ± 3.1	18.3 ± 3.8
*S. aureus*	49.2 ± 1.7	40.2 ± 2.9
*B. cereus*	21.8 ± 2.4	20.4 ± 1.5

**Table 6:** Antioxidant activity of cell-free extract isolated from *L. reuteri*LR12.

Sample (µg/mL)	DPPH Scavenging	Hydroxyl Radical	Superoxide Radical	Reducing Power
12.5	28 ± 1.3	33 ± 2.3	6.2 ± 0.56	14.2 ± 1.5
25	33 ± 2.9	43 ± 2.4	38.4 ± 1.5	30.5 ± 1.1
50	53 ± 3.8	51 ± 3.2	52.3 ± 4.3	56.4 ± 3.2
75	87 ± 2.7	76 ± 1.9	79.6 ± 3.9	80.2 ± 2.8
100	89 ± 2.1	80 ± 2.2	89.2 ± 2.1	90.4 ± 1.7

**Table 7:** Antioxidant activity of cell free extract isolated from *L. lactis*LL10.

Sample (µg/mL)	DPPH Scavenging	Hydroxyl Radical	Superoxide Radical	Reducing Power
12.5	15 ± 1.5	20 ± 3.9	28 ± 3.4	12.4 ± 3.2
25	69 ± 2.1	44 ± 2.8	31 ± 2.5	20.4 ± 1.9
50	78 ± 5.3	53 ± 1.6	48 ± 3.3	49.2 ± 2.3
75	81 ± 1.5	89 ± 2.7	78 ± 2.1	75.3 ± 3.4
100	90 ± 3.7	90 ± 3.3	84 ± 1.6	86.5 ± 1.7

**Table 8:** Microbial population in yogurt during storage.

Microorganisms	Treatment		
	1% Puree	2% Puree	3% Puree
Coliforms (CFU/mL)	ND	ND	ND
Total bacteria (×10^−8^ CFU/mL)	2.81	1.32	1.03
Fungi (×10^−2^ CFU/mL)	15	13	10
